# Impact of on-pump and off-pump coronary artery bypass grafting on 10-year mortality versus percutaneous coronary intervention

**DOI:** 10.1093/ejcts/ezad240

**Published:** 2023-06-22

**Authors:** Shigetaka Kageyama, Patrick W Serruys, Kai Ninomiya, Neil O’Leary, Shinichiro Masuda, Nozomi Kotoku, Antonio Colombo, Robert-Jan van Geuns, Milan Milojevic, Michael J Mack, Alan Soo, Scot Garg, Yoshinobu Onuma, Piroze M Davierwala, SYNTAX Extended Survival Investigators, Filip Casselman, Bernard de Bruyne, Evald Høj Christiansen, Juan M Ruiz-Nodar, Paul Vermeersch, Werner Schultz, Manel Sabaté, Giulio Guagliumi, Herko Grubitzsch, Karl Stangl, Olivier Darremont, M Bentala, Peter den Heijer, Istvan Preda, Robert Stoler, Michael J Mack, Tamás Szerafin, John K Buckner, Myles S Guber, Niels Verberkmoes, Ferdi Akca, Ted Feldman, Friedhelm Beyersdorf, Benny Drieghe, Keith Oldroyd, Geoff Berg, Anders Jeppsson, Kimberly Barber, Kevin Wolschleger, John Heiser, Pim van der Harst, Massimo A Mariani, Hermann Reichenspurner, Christoffer Stark, Mika Laine, Paul C Ho, John C Chen, Richard Zelman, Phillip A Horwitz, Andrzej Bochenek, Agata Krauze, Christina Grothusen, Dariusz Dudek, George Heyrich, Piroze Davierwala, Thilo Noack, Philippe Kolh, Victor LeGrand, Pedro Coelho, Stephan Ensminger, Boris Nasseri, Richard Ingemansson, Goran Olivecrona, Javier Escaned, Reddy Guera, Sergio Berti, Marie-Claude Morice, Alaide Chieffo, Nicholas Burke, Michael Mooney, Alvise Spolaor, Christian Hagl, Michael Näbauer, Maarten Jan Suttorp, Ronald A Stine, Thomas McGarry, Scott Lucas, Knut Endresen, Andrew Taussig, Kevin Accola, Umberto Canosi, Ivan Horvath, Louis Cannon, John D Talbott, Chris W Akins, Robert Kramer, Michael Aschermann, William Killinger, Inga Narbute, David R Holmes, Francesco Burzotta, Ad Bogers, Felix Zijlstra, Helene Eltchaninoff, Jacques Berland, Giulio Stefanini, Ignacio Cruz Gonzalez, Uta Hoppe, Stefan Kiesz, Bartlomiej Gora, Anders Ahlsson, Matthias Corbascio, Thomas Bilfinger, Didier Carrie, Didier Tchétché, Karl-Eugen Hauptman, Elisabeth Stahle, Stefan James, Sigrid Sandner, Günther Laufer, Irene Lang, Adam Witkowski, Vinod Thourani, Harry Suryapranata, Simon Redwood, Charles Knight, Philip MacCarthy, Nick Curzen, Adam de Belder, Adrian Banning, Anthony Gershlick

**Affiliations:** Department of Cardiology, National University of Ireland, Galway (NUIG), Galway, Ireland; Department of Cardiology, National University of Ireland, Galway (NUIG), Galway, Ireland; National Heart and Lung Institute, Imperial College London, London, UK; Department of Cardiology, National University of Ireland, Galway (NUIG), Galway, Ireland; Department of Cardiology, National University of Ireland, Galway (NUIG), Galway, Ireland; Department of Cardiology, National University of Ireland, Galway (NUIG), Galway, Ireland; Department of Cardiology, National University of Ireland, Galway (NUIG), Galway, Ireland; Department of Biomedical Sciences, Humanitas University, Pieve Emanuele, Milan, Italy; Humanitas Clinical and Research Centre IRCCS, Milan, Italy; Department of Cardiology, Radboud University Medical Centre, Nijmegen, Netherlands; Department of Cardiothoracic Surgery, Erasmus University Medical Centre, Rotterdam, Netherlands; Department of Cardiac Surgery and Cardiovascular Research, Dedinje Cardiovascular Institute, Belgrade, Serbia; Department of Cardiothoracic Surgery, Baylor University Medical Center, Dallas, TX, USA; Department of Cardiothoracic Surgery, University Hospital Galway, Galway, Ireland; Department of Cardiology, Royal Blackburn Hospital, Blackburn, UK; Department of Cardiology, National University of Ireland, Galway (NUIG), Galway, Ireland; Department of Surgery, University of Toronto, Toronto, ON, Canada; Division of Cardiovascular Surgery, Peter Munk Cardiac Centre, Toronto General Hospital, Toronto, ON, Canada; University Health Network, Toronto, ON, Canada

**Keywords:** Coronary artery bypass grafting, Revascularization, Complex coronary artery disease, Percutaneous coronary intervention, Ten-year mortality

## Abstract

**OBJECTIVES:**

The very long-term mortality of off-pump and on-pump coronary artery bypass grafting (CABG) versus percutaneous coronary intervention (PCI) in a randomized complex coronary artery disease population is unknown. This study aims to investigate the impact of on-pump and off-pump CABG versus PCI on 10-year all-cause mortality.

**METHODS:**

The SYNTAX trial randomized 1800 patients with three-vessel and/or left main coronary artery disease to PCI or CABG and assessed their survival at 10 years. In this sub-study, the hazard of mortality over 10 years was compared according to the technique of revascularization: on-pump CABG (*n* = 725), off-pump CABG (*n* = 128) and PCI (*n* = 903).

**RESULTS:**

There was substantial inter-site variation in the use of off-pump CABG despite baseline characteristics being largely homogeneous among the 3 groups. The crude rate of mortality was significantly lower following on-pump CABG versus PCI [25.6% vs 28.4%, hazard ratio (HR) 0.79, 95% confidence interval (CI) 0.65–0.96], while it was comparable between off-pump CABG and PCI (28.5% vs 28.4%, HR 0.98, 95% CI 0.69–1.40). After adjusting for the 9 variables included in the SYNTAX score II 2020, 10-year mortality remained significantly lower with on-pump CABG than PCI (HR 0.75 against PCI, *P* = 0.009).

**CONCLUSIONS:**

In the SYNTAXES trial, 10-year mortality adjusted for major confounders was significantly lower following on-pump CABG compared to PCI. There was no evidence for unadjusted difference between off-pump CABG and PCI, although the unadjusted estimated HR had a wide CI. Site heterogeneity in the technique used in bypass surgery has had measurable effects on treatment performance.

**Figure:**
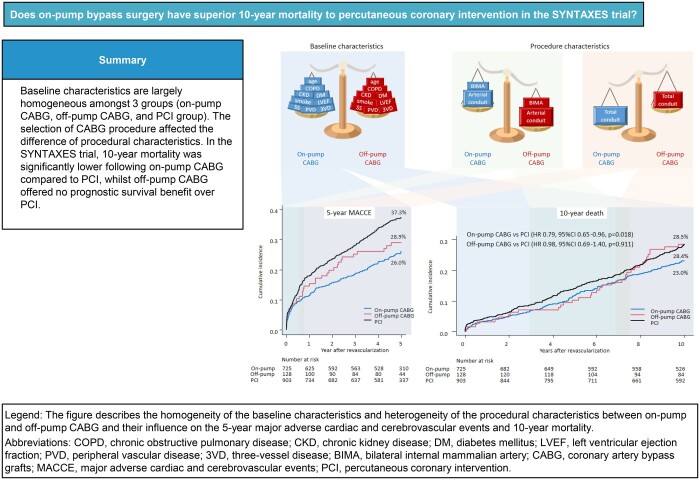

## INTRODUCTION

For 30 years, the risks and benefits of the off-pump versus on-pump approach to coronary artery bypass grafting (CABG) surgery have been debated extensively [1]. CABG using cardiopulmonary bypass (CPB) (on-pump) is both a safe and effective way to revascularize complex coronary artery disease (CAD); however, it is associated with a higher incidence of perioperative complications, and in an effort to address this, CABG without using CPB (off-pump) was introduced into daily practice widely. The systematic meta-analysis from randomized control trial showed that the incidence of the major adverse cardiac and cerebrovascular events did not differ with or without CPB for the longest available follow-up, while on-pump surgery was associated with an increased occurrence of stroke, renal impairment and mediastinitis. The incidence of mid-term graft failure and the need for repeat revascularization was increased after off-pump surgery [2].

A meta-analysis including 20 627 patients showed a higher risk of mortality in off-pump versus on-pump CABG [3], with the main reasons lower rates of complete revascularization and reduced graft patency, mainly when off-pump surgery is performed by surgeons with limited experience [4–6].

Randomized controlled trials and large observational studies focusing on three-vessel disease (3VD) have uniformly demonstrated that CABG is associated with significantly lower mortality than percutaneous coronary intervention (PCI) [7, 8]. The SYNTAXES trial, aiming for extended follow-up of the SYNTAX trial population from 5 to 10 years, showed no significant difference in all-cause death between PCI and CABG at 10 years, while CABG provided a significant survival benefit in patients with 3VD [9, 10]. However, the specific implications of off-pump versus on-pump surgery relative to PCI are still to be determined. Thus, we focused on the impact of on-pump and off-pump CABG versus PCI on very long-term all-cause mortality.

## METHODS

### Ethical statement

In the main trial, written informed consent was taken by each participating centre and the study was approved by the institutional review board (MEC-2016-716).

### Study design

The design and the primary results of the SYNTAX study have been reported previously [9, 10]. Briefly, all-comer patients with *de novo* 3VD and/or left main CAD were enrolled and randomized to either CABG (*n* = 897) or PCI (*n* = 903) with paclitaxel-eluting stent (TAXUS, Boston Scientific, Marlborough, MA, USA). The SYNTAXES study (NCT03417050) was an investigator-driven extended follow-up which aimed to evaluate vital status at up to 10 years.

All the baseline, periprocedural and post-procedural characteristics were extracted from the electronic clinical report form. For the purposes of these analyses, the 3 comparator revascularization groups in this patient cohort were on-pump CABG, off-pump CABG and PCI, as per the intention to treat. Each group's cumulative mortality of up to 10 years was analysed with and without adjustment for baseline characteristics. Notably, since the procedural technique was left to the operator’s discretion, significant inter-institutional and regional disparities in the presence and absence of a pump during CABG were expected. Sites were grouped by country and using the United Nations geoscheme for regions to help assess the heterogeneous use of on- and off-pump CABG [11].

### Study end points

The primary end point was time to all-cause death over 10 years. Vital status was confirmed by contact with medical personnel, electronic healthcare record review or municipal survival record.

### Statistical analysis

For descriptive summaries, categorical variables are expressed as numbers and percentages, and continuous variables are median [1st and 3rd interquartile range]. To compare the specific 2 groups to be focused on, unpaired *t*-tests for continuous variables and Fisher’s exact test or chi-square test for categorical variables. A 95% confidence interval (CI) of odds ratio for the categorical values or that of mean differences between groups for continuous values was presented.

The Kaplan–Meier estimator was used to assess unadjusted and adjusted time to 10-year mortality. The treatment effect of on-pump and off-pump CABG against PCI is presented as a hazard ratio (HR) with a 95% CI. After presenting crude data, the inverse probability of treatment weight (IPTW) adjustment based on the 9 baseline factors included in SYNTAX score II 2020 was done for the comparison between on-pump CABG versus PCI [12, 13]. Those who have missing value(s) concerning 9 factors were removed from IPTW adjustment. The detailed methods are described in the Supplementary Material. IPTW adjustment was confirmed by the sensitivity analysis described also in the Supplementary Material. We did not perform the same adjustment analysis for off-pump CABG versus PCI because of the instability of the model from a statistical point of view.

All statistical analyses were performed using R 4.1.1 (The R Foundation for Statistical Computing, Vienna, Austria). All reported *P* < 0.05 was considered statistically significant. Either the CI of odds ratio does not contain 1 or the CI of difference between groups does not contain 0, the compared 2 groups are recognized to have a significant difference.

## RESULTS

### Baseline characteristics among the 3 groups

**Figure 1:**
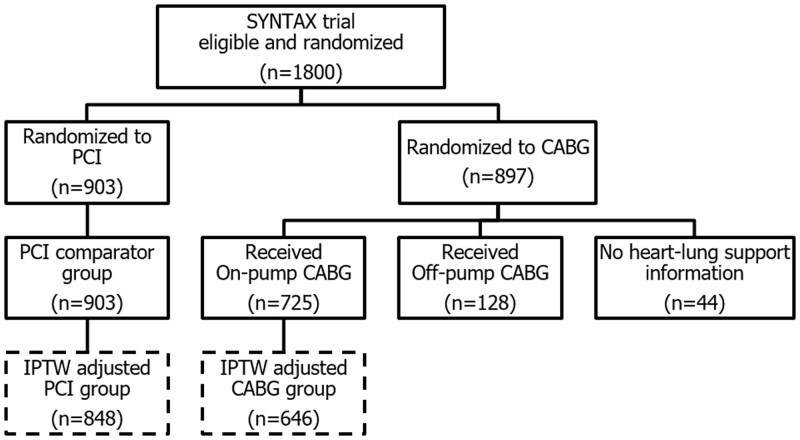
Study flowchart illustration. The sample size for the comparator groups analysed for the primary analysis was reduced slightly due to missingness in a small proportion of baseline variables used in the adjusted model. CABG: coronary artery bypass grafts; PCI: percutaneous coronary intervention.

**Figure 2:**
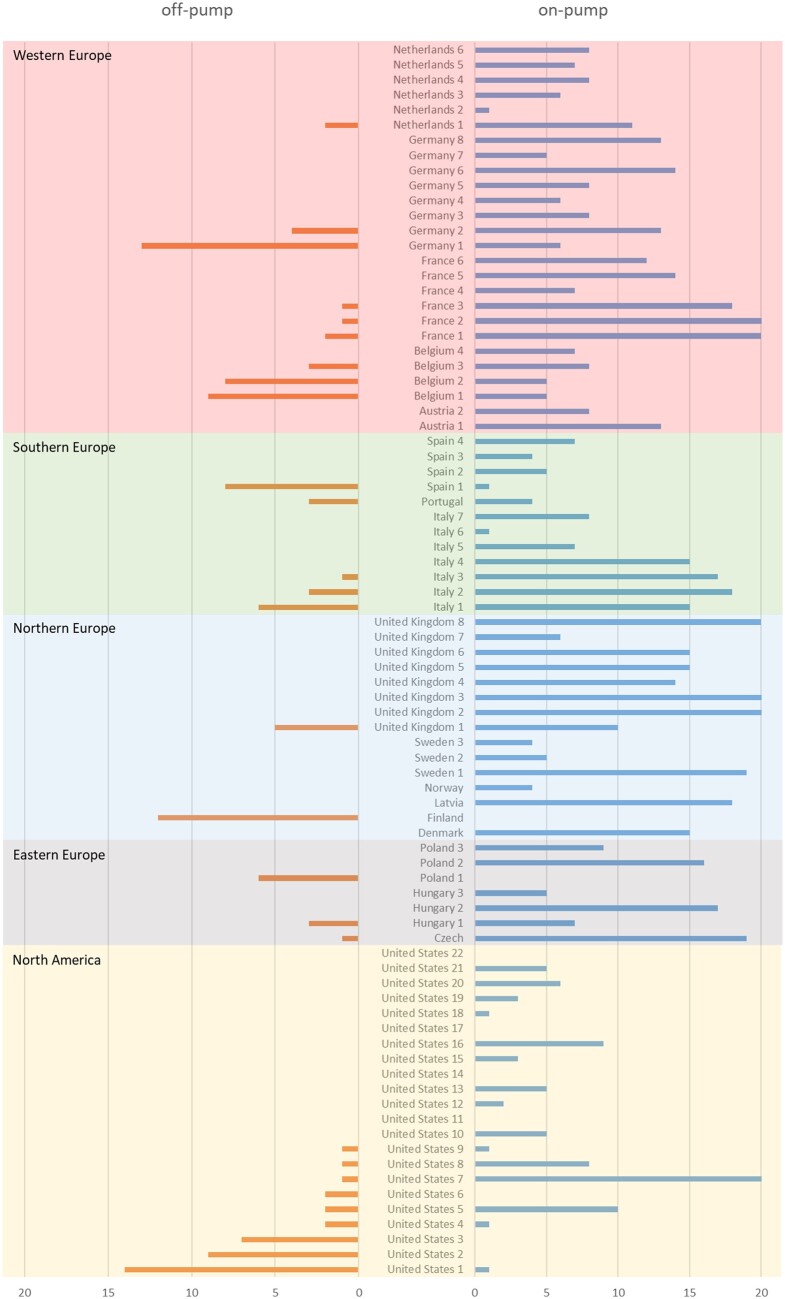
Region disparity of on-pump and off-pump in SYNTAX trial. Patient number of the on-pump and off-pump CABG per institute stratified by country and region. In 4 centres with only 1 or 2 patients recruited, there was no allocation to the CABG arm as per randomization. CABG: coronary artery bypass graft.

The randomized cohort of the SYNTAX study enrolled 1800 patients from 18 countries, of whom 5 did not participate in the 10-year follow-up and were censored at 5 years. Of the 897 patients in the CABG randomized arm, 44 did not have intraoperative information regarding the type of heart–lung support and were excluded from analysis in this sub-study (Fig. 1). The median follow-up in the full cohort was 11.2 (7.7, 12.1) years. There was a heterogeneous distribution of off-pump CABG procedures across centres (Fig. 2), with it performed in only 28 of the 85 participating institutions (32.9%), with 5 centres performing it exclusively. In 4 centres with only 1 or 2 patients recruited, there was no allocation to the CABG arm as per randomization.

Baseline characteristics grouped according to the revascularization strategies are shown in Table 1. The 9 prognostic determinants of the SYNTAX score II 2020 were evenly distributed among the 3 groups except for smoking habit (ever smoker). The rate of hypertension was the highest in the PCI group (68.9%), while the rate of patients with anaemia at baseline was the highest in the off-pump CABG group (37.4%).

**Table 1:** Baseline patient characteristics

Factor	Group	On-pump, *N* = 725	Off-pump, *N* = 128	PCI, *N* = 903
*Age*		66 (33, 86)	65 (32, 85)	66 (36, 89)
Sex, *n* (%)	Male	580 (80.0)	98 (76.6)	690 (76.4)
	Female	145 (20.0)	30 (23.4)	213 (23.6)
Region, *n* (%)	North America	79 (10.9)	37 (28.9)	123 (13.6)
	Eastern Europe	73 (10.1)	10 (7.8)	97 (10.7)
	Northern Europe	188 (25.9)	17 (13.3)	214 (23.7)
	Southern Europe	102 (14.1)	21 (16.4)	134 (14.8)
	Western Europe	283 (39.0)	43 (33.6)	335 (37.1)
Caucasian, *n* (%)		696 (96.0)	121 (94.5)	875 (96.9)
Procedure status, *n* (%)	Elective	671 (92.6)	116 (90.6)	833 (94.1)
	Urgent	54 (7.4)	12 (9.4)	52 (5.9)
BMI		27.0 (24.7, 30.2)	28.3 (25.2, 30.9)	27.4 (24.9, 30.9)
CKD (%)		120 (18.4)	28 (23.3)	167 (19.6)
*CrCl (ml/min)*		81.7 (65.0, 103.1)	85.4 (61.7, 102.7)	80.0 (64.2, 103.3)
Anemia, *n* (%)		176 (28.7)	43 (37.4)	205 (25.4)
Hb (g/dl)		13.8 (12.6, 14.8)	13.7 (12.2, 14.7)	14.0 (12.9, 14.9)
*Ever smoker, n (%)*		153 (21.2)	35 (27.6)	167 (18.5)
*COPD, n (%)*		71 (9.8)	7 (5.5)	71 (7.9)
Dyslipidemia, *n* (%)		565 (78.7)	91 (71.7)	705 (78.7)
LDL-cholesterol (mg/dl)		87.4 (66.5, 112.4)	88.4 (68.0, 114.8)	93.3 (73.5, 117.6)
CRP (mg/dl)		0.34 (0.13, 1.48)	0.42 (0.18, 1.14)	0.33 (0.13, 0.87)
*Medically treated DM, n (%)*		169 (23.3)	32 (25.0)	231 (25.6)
Insulin use, *n* (%)		71 (9.8)	14 (10.9)	89 (9.9)
HbA1c %		5.9 (5.5, 6.5)	5.9 (5.6, 6.4)	5.9 (5.6, 6.4)
Hypertension, *n* (%)		466 (64.3)	76 (59.4)	622 (68.9)
Metabolic syndrome, *n* (%)		253 (44.5)	58 (50.9)	339 (46.0)
EF < 50%, *n* (%)		150 (20.7)	23 (18.0)	193 (21.4)
*LVEF %*		60.0 (50.0, 65.5)	59.0 (50.0, 66.0)	60.0 (52.0, 67.0)
Pre-HF, *n* (%)		37 (5.2)	7 (5.6)	36 (4.0)
Pre-CEVD, *n* (%)		106 (14.7)	21 (16.4)	119 (13.2)
Pre-stroke, *n* (%)		34 (4.7)	7 (5.5)	35 (3.9)
Pre-MI, *n* (%)		248 (34.4)	35 (28.5)	285 (31.9)
*Pre-PVD, n (%)*		76 (10.5)	14 (10.9)	82 (9.1)
Disease type, *n* (%)	3VD	445 (61.4)	72 (56.2)	546 (60.5)
	LMCAD	280 (38.6)	56 (43.8)	357 (39.5)
Bifurcation lesion, *n* (%)		540 (74.8)	81 (64.3)	649 (72.4)
Total occlusion, *n* (%)		153 (21.2)	36 (28.6)	217 (24.2)
Number of lesions		4 (3, 6)	4 (3, 5)	4 (3, 5)
*Anatomical SYNTAX score*		28.0 (21.0, 37.0)	27.5 (18.0, 35.0)	27.0 (20.0, 35.0)
Silent ischaemia, *n* (%)		100 (13.8)	20 (15.6)	127 (14.1)
Stable angina, *n* (%)		419 (57.8)	72 (56.2)	514 (56.9)
Unstable angina, *n* (%)		206 (28.4)	36 (28.1)	262 (29.0)
IABP use, *n* (%)		22 (3.0)	3 (2.3)	43 (4.8)
SF36-MCS		47.6 (37.4, 55.1)	43.8 (35.4, 53.9)	46.1 (36.0, 55.2)
SF36-PCS		41.5 (33.1, 48.5)	39.4 (31.4, 47.5)	39.9 (32.7, 47.6)
Logistic EuroSCORE		4.00 (2.00, 5.00)	3.00 (2.00, 5.25)	4.00 (2.00, 5.00)
SYNTAX score II 2020		20.0 (11.0, 33.0)	19.0 (11.0, 34.0)	24.0 (14.0, 39.0)

Factors highlighted in italic are related to the SYNTAX score II 2020 and used for IPTW adjustment. Values are represented as median (1st, 3rd interquartile range). The number (proportion) of missing data values for each variable is as follows; BMI 1 (0.1%), creatinine clearance 132 (7.5%), Hb 220 (12.5%), LDL-cholesterol 203 (11.6%), CRP 123 (7.0%), HbA1c 212 (12.1%), number of lesions 11 (0.6%), anatomical SYNTAX score 9 (0.6%), SF36-MCS 136 (7.7%), SF36-PCS 136 (7.7%), CKD 132 (7.5%), ever smoker 5 (0.3%), dyslipidemia 15 (0.9%), metabolic syndrome 336 (19.1%), LVEF (categorical) 28 (1.6%), pre-HF 21 (1.2%), pre-CEVD 8 (0.5%), pre-stroke 8 (0.5%), pre-MI 20 (1.1%), bifurcation lesion 11 (0.6%), TO 11 (0.6%) and IABP 7 (0.4%).

BMI: body mass index; CEVD: cerebrovascular disease; COPD: chronic obstructive pulmonary disease; CrCl: creatinine clearance; CRP: C-reactive protein; DM: diabetes mellitus; EF: ejection fraction; Hb: haemoglobin; HbA1c: glycated haemoglobin; LDL: low-density lipoprotein; HF: heart failure; IABP: intra-aortic balloon pump; LMCAD: left main coronary artery disease; LVEF: left ventricular ejection fraction; MCS: mental component summary; MI: myocardial infarction; PCI: percutaneous coronary intervention; PCS: physical component summary; PVD: peripheral vascular disease; SF: short form; 3VD: three-vessel disease.

When comparing on-pump and off-pump CABG, there were no significant differences in comorbidities or lesion complexity as semi-quantified by the anatomic SYNTAX score, even though bifurcation lesions were significantly more frequent in the on-pump CABG group (74.8% vs 64.3%, 95% CI 0.40–0.93). Mental and physical status per the SF-36 were comparable, as were rates of emergent cases, angina status and the logistic EuroSCORE. Notably, the estimated 10-year mortality calculated by the SYNTAX score II 2020 was highest in the PCI group, with no significant difference between the on-pump and off-pump CABG groups.

### Differences in procedural characteristics of on-pump and off-pump CABG

The procedural characteristics are presented in Table 2. Patients in the on-pump CABG group had a significantly higher total number of conduits compared to the off-pump CABG group (3 [2, 3] vs 3 [2, 3], 95% CI 0.06–0.33). The left internal mammary artery (IMA) was used in >95% of patients in both groups, however, usage of the right IMA and bilateral IMAs was significantly lower in the on-pump versus the off-pump group (26.5% vs 36.7%, *P* = 0.019, 26.1% vs 36.7%, *P* = 0.018, respectively). The number of arterial conduits per patient was higher in the off-pump group (1 [1, 2] vs 1 [1, 2], 95% CI −0.27 to −0.03), while the use of venous conduits (1 [1, 2] vs 1 [0, 2], 95% CI 0.17–1.07) was significantly higher in the on-pump group. The usage of jump grafts was similar (31.2%). Operation times were significantly longer with on-pump versus off-pump CABG (205 [165, 245] vs 183 [156, 225] min, 95% CI 5.44–29.9).

**Table 2:** Procedural characteristics

Factor	On-pump	Off-pump	95% CI
*N*	725	128	
IABP use (%)	22 (3.0)	3 (2.3)	0.14 to 2.61
Cardioplegia, *n* (%)	690 (95.2)	0	
Hypothermia, *n* (%)	585 (80.7)	0	
Complete revascularization, *n* (%)	464 (64.0)	81 (63.3)	0.65 to 1.47
Incomplete revascularization in inferolateral wall, *n* (%)	205 (28.8)	43 (33.6)	0.81 to 1.90
Bilateral IMA use, *n* (%)	189 (26.1)	47 (36.7)	1.08 to 2.48
Left IMA use, *n* (%)	703 (97.0)	127 (99.2)	0.63 to 165.2
Right IMA use, *n* (%)	192 (26.5)	47 (36.7)	1.06 to 2.43
Jump graft use, *n* (%)	225 (31.2)	40 (31.2)	0.65 to 1.53
Number of lesions	4 (3, 6)	4 (3, 5)	−0.03 to 0.65
Number of total conduits	3 (2, 3)	3 (2, 3)	0.06 to 0.33
Number of arterial conduits	1 (1, 2)	1 (1, 2)	−0.27 to −0.03
Number of venous conduits	1 (1, 2)	1 (0, 2)	0.17 to 1.07
Operation time (min)	205 (165, 245)	183 (156, 225)	5.44 to 29.9

Values are represented as median (1st, 3rd interquartile range). To compare the difference between 2 groups, a 95% CI of odd’s ratio for the categorical values or that of mean differences between groups for continuous values was presented. The number (proportion) of missing data values for each variable is as follows: number of lesions 5 (0.6%), number of total conduits 1 (0.1%) and complete revascularization 13 (0.5%).

CI: confidence interval; IABP: intra-aortic balloon pump; IMA: internal mammalian artery.

In the CABG group, the rate of complete revascularization, which was reported according to the postoperative surgical report incorporated into the anatomic SYNTAX score (refer to SYNTAX Score surgical), was similar between on-pump and off-pump CABG (64% vs 63.3%). Incomplete revascularization of the right coronary artery and left circumflex, which perfuses the inferolateral wall, was numerically higher but not statistically significant with off-pump than on-pump CABG (33.6% vs 28.8%, 0.81–1.90).

Peri procedural cerebrovascular events were reported in 13 cases (1.8%) in the on-pump CABG group, while none occurred with off-pump CABG.

### Ten-year mortality after revascularization

**Figure 3:**
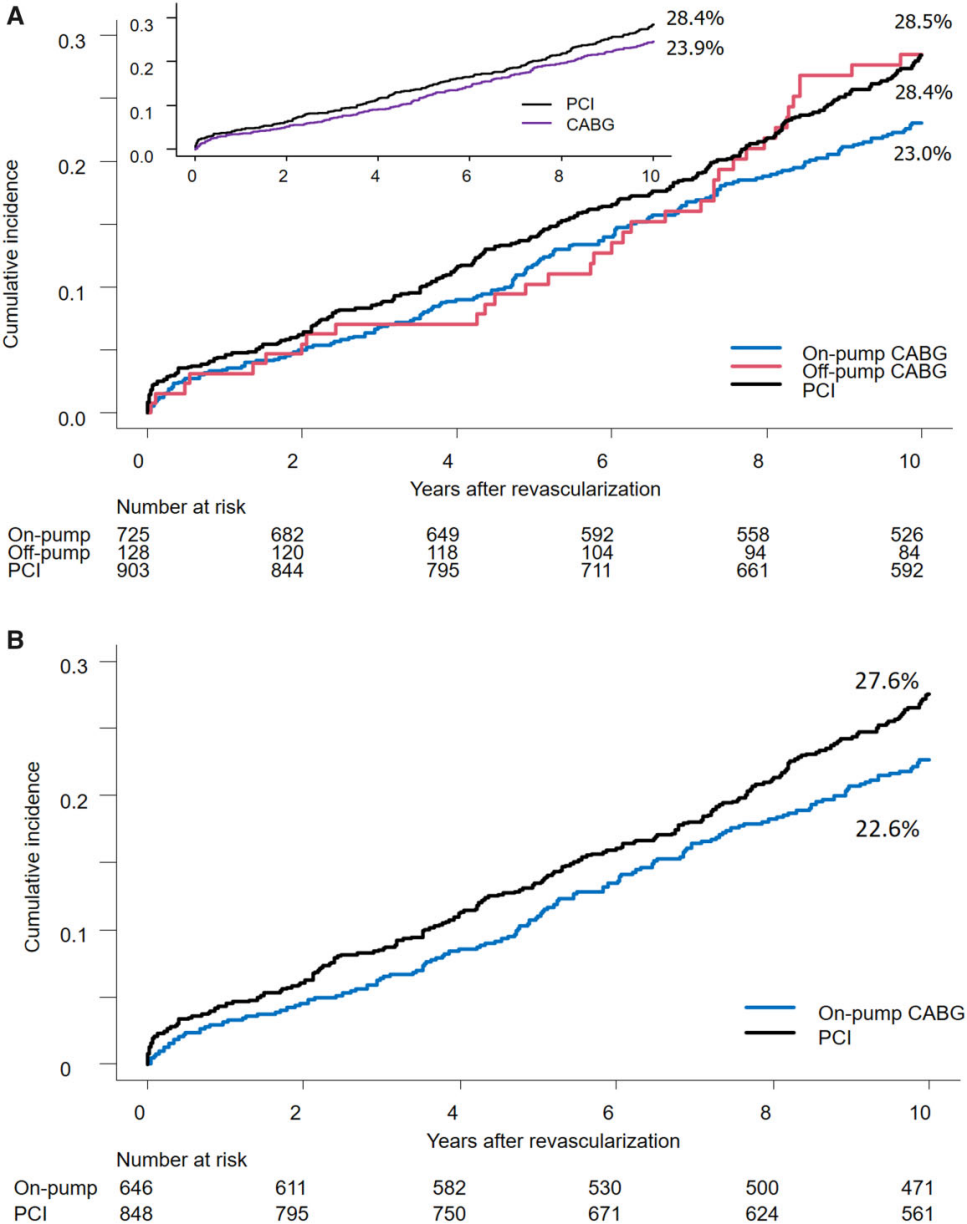
Crude and adjusted survival curve of 10-year mortality. (**A**) Crude survival curve of 10-year mortality. Image (**A**) shows rude Kaplan–Meier mortality curves in 3 groups (on-pump CABG, off-pump CABG and PCI). (**B**) Inverse probability weighting-adjusted survival curves of on-pump CABG and PCI. Image (**B**) shows inverse probability weighting-adjusted survival curves of on-pump CABG and PCI. CABG: coronary artery bypass grafts; COPD: chronic obstructive pulmonary disease; EF: ejection fraction; PCI: percutaneous coronary intervention.

As presented in Fig. 3A, crude cumulative 10-year mortality after revascularization was lowest with on-pump CABG (23.0%), while rates after off-pump CABG (28.4%) and PCI (28.5%) were comparable. Crude mortality stratified by anatomical SYNTAX score is presented in Supplementary Material, Fig. S1. Cox regression analysis of 10-year mortality showed a significant risk reduction with on-pump CABG versus PCI (HR 0.79, 95% CI 0.65–0.96, *P* = 0.018) while no significant difference was seen between off-pump CABG and PCI (HR 0.98, 95% CI 0.69–1.40, *P* = 0.911). The mortality rates at 5 years post-revascularization were 10.2%, 11.7% and 14% with off-pump CABG, on-pump CABG and PCI, respectively. However, no significant differences were seen in Cox regression analysis at 5 years between off-pump and PCI (HR 0.68, 95% CI 0.36–1.25) or on-pump and PCI (HR 0.79, 95% CI 0.60–1.06).

After IPTW adjustment by the factors included in the SYNTAX score II 2020, on-pump CABG had lower 10-year mortality compared to PCI (HR 0.75, 95% CI 0.62–0.93, *P* = 0.009, Fig. 3B). A total of 134 patients having more than 1 missing value were removed from the IPTW adjustment. The sensitivity analysis confirmed this superiority after considering the additional confounding factors: sex, hypertension and the CABG procedure heterogeneity among sites (Supplemental Material).

## DISCUSSION

The main findings of this study are:

- There is a huge disparity in the selection of on-pump and off-pump CABG among sites, as well as countries and regions.
- The cumulative incidence of crude 10-year mortality was significantly lower with on-pump CABG compared to PCI, and not different between off-pump CABG and PCI.
- The differences between on-pump CABG and PCI in 10-year mortality remained significant after adjustment for the prognostic factors incorporated into the SYNTAX score II 2020.

This is the first report on the impact of on-pump CABG compared to PCI on 10-year mortality in a complex CAD population randomized to either CABG or PCI.

First, it is imperative to highlight the disparity in the selection of on-pump and off-pump CABG among sites, especially as this potentially confounding factor on outcomes has never previously been examined in the SYNTAXES trial. During the systematic review of previously published regional differences in the SYNTAXES trial, we first recognized the disparity of treatment methods in the CABG group [14]. It is also imperative to question whether this discrepancy is also seen in other randomized trials of complex CAD, since it could influence the interpretation of the treatment effect of CABG against PCI. Consequently, it is paramount to aggregate similar randomized trials to establish whether this observation is confirmed in other trials.

Numerous patient and clinical factors influence the decision to perform a CABG operation on-pump versus off-pump [4, 5]. Since the surgical technique was left to the surgeon's discretion, we genuinely expected frailer patients with comorbidities such as chronic obstructive lung disease and cerebrovascular disease to be over-represented in the off-pump group, with patients having more complex anatomy predominating in the on-pump group. In reality, however, there were no significant differences in the SYNTAX score or logistic EuroSCORE between the 2 surgical approaches. Similarly, the mental and physical component summary of the SF 36 self-report questionnaire, a reliable evaluation of frailty and mental condition at baseline, was also comparable [15]. In terms of specific outcomes, the prevention of cerebrovascular complications (1.8% vs 0%) was indeed achieved by the aortic ‘no-touch technique’ and remains one of the most important motivations for using the off-pump technique.

Somewhat paradoxical, but interesting observations on procedural characteristics are reported. The total number of conduits was higher in the on-pump group, reflecting the numerically greater number of lesions. However, another plausible explanation may be that surgeons working on-pump had better technical opportunities to bypass target vessels that were located along the inferior and posterior walls; incomplete revascularization rate was higher in off-pump cases, even though jump grafts were used equally. Of note, bilateral IMAs are used more frequently in the off-pump group. Taggart *et al.* reported that 10-year mortality was comparable in 2 groups assigned bilateral versus single IMA (20.3% vs 21.2%) [16]. The on-pump and off-pump procedures were performed in equal measures in both arms, while the number of patients having >3 vessels grafted was lower with bilateral IMAs. Ultimately, there is a trade-off between better long-term graft patency with arterial conduits and lower complete revascularization rates; the merits of bilateral IMAs might have been offset by relatively higher incomplete revascularization.

The crude survival curve after off-pump CABG changes around the seventh year of follow-up. Eventually, after 10 years, it becomes identical to the PCI group. Of note, at 5 years, the crude mortality in the off-pump group was comparable to the on-pump group, which is at variance with the EXCEL trial, which reported diverging rates of 3-year mortality after IPTW adjustment of 8.8% and 4.5% with off-pump versus on-pump CABG, respectively [17].

Our findings are both concordant and discordant with the results of the 10-year follow-up of the ROOBY trial, which was conducted in the same period as the SYNTAX trial and randomized 2203 patients to off-pump CABG and on-pump CABG [18]. Both trials reported lower 10-year mortality with on-pump versus off-pump CABG; in SYNTAXES, mortality with off-pump CABG was lower than on-pump CABG between 4 and 6 years after revascularization, while in the ROOBY trial mortality with on-pump CABG was consistently lower than off-pump throughout the trial. The overall 10-year CABG mortality was lower in SYNTAXES (24%) than in ROOBY (32.7%) despite the population in SYNTAX being on average 2 years older. Presumably, the participating sites in SYNTAX selected the most appropriate procedure for each patient.

Not only preprocedural and periprocedural aspects but also post-procedural factors affect long-term mortality. Medications at discharge and 5 years after the index procedure are presented in the Supplementary Material, which shows non-inferior medications had been done in the off-pump CABG and PCI arm compared to the on-pump CABG arm.

We demonstrated that on-pump CABG had a significantly low incidence of 10-year mortality compared to PCI after adjustment. However, due to the small number of off-pump CABG procedures, we could not apply the same statistical approach to compare off-pump CABG to PCI. It would be desirable to pool randomized trials to overcome this issue of underpowering.

### Limitations

The current study has several limitations. First, the present study on on-pump and off-pump CABG compared to PCI was not randomized and is a post hoc analysis. All reported findings should be considered exploratory and hypothesis generating. Second, the SYNTAX trial was conducted between 2005 and 2007 with the predominant use of first-generation PES in the PCI arm, which may limit the generalizability of our findings to current clinical practice. Conversely, CABG procedures have improved as well as its outcome. It is, however, unavoidable that the findings from long-term follow-up data are based on outdated technology while the evidence for contemporary technology can be derived only from short-term follow-up studies. Third, heterogeneity of the CABG procedure was substantial among sites, countries and regions. IPTW adjustment even carefully applied may not have overcome all the confounding factors. Fourth, the SYNTAXES study was performed to evaluate survival for up to 10 years, and the end point was all-cause death only. We do not have the data on MACCE beyond 5 years. However, the SYNTAXES study provides the first randomized trial that was meticulously collected and achieved a high follow-up rate of 93.8% for 10-year vital status (1689 of 1800 enrolled patients). Even though the overall sample size was sufficient and the 10-year follow-up rate was high enough for the primary purpose of the SYNTAXES trial, the subdivision of the global cohort into 3 groups resulted in insufficient statistical power for comparing off-pump to PCI or on-pump CABG.

It is desirable to conduct an additional investigation on this topic using large patient-based analyses with very long-term follow-up using a pooled dataset coming from randomized trials assigning CABG and PCI in complex CAD [8, 19, 20].

## CONCLUSIONS

In the SYNTAXES trial, 10-year mortality adjusted for major confounders was significantly lower following on-pump CABG compared to PCI, whereas the unadjusted difference between off-pump CABG and PCI was a crude analysis with no confounders control and the unadjusted estimated HR had a wide CI. Site heterogeneity in the technique used in bypass surgery could have affected treatment performance. Given its impact on outcomes, it should be pre-stratified in future trials.

## Supplementary Material

ezad240_Supplementary_Data

## Data Availability

The data underlying this article will be shared on reasonable request to the corresponding author.

## ABBREVIATIONS

3VD: Three-vessel disease
CABG: Coronary artery bypass grafting
CAD: Coronary artery disease
CI: Confidence interval
CPB: Cardiopulmonary bypass
HR: Hazard ratio
IMA: Internal mammary artery
IPTW: Inverse probability of treatment weight
PCI: Percutaneous coronary intervention

## References

[1] Gaudino M , Angelini GD, Antoniades C, Bakaeen F, Benedetto U, Calafiore AM et al Off-pump coronary artery bypass grafting: 30 years of debate. J Am Heart Assoc 2018;7:e009934. 30369328 10.1161/JAHA.118.009934 PMC6201399

[2] Deppe AC , Arbash W, Kuhn EW, Slottosch I, Scherner M, Liakopoulos OJ et al Current evidence of coronary artery bypass grafting off-pump versus on-pump: a systematic review with meta-analysis of over 16 900 patients investigated in randomized controlled trials. Eur J Cardiothorac Surg 2016;49:1031–41; discussion 1041. 26276839 10.1093/ejcts/ezv268

[3] Gaudino M , Benedetto U, Bakaeen F, Rahouma M, Tam DY, Abouarab A et al Off- versus on-pump coronary surgery and the effect of follow-up length and surgeons' experience: a meta-analysis. J Am Heart Assoc 2018;7:e010034. 30373421 10.1161/JAHA.118.010034 PMC6404195

[4] Shroyer AL , Hattler B, Wagner TH, Collins JF, Baltz JH, Quin JA et al; Veterans Affairs ROOBY-FS Group. Five-year outcomes after on-pump and off-pump coronary-artery bypass. New Engl J Med 2017;377:623–32. 28813218 10.1056/NEJMoa1614341

[5] Lamy A , Devereaux PJ, Prabhakaran D, Taggart DP, Hu SS, Straka Z et al; CORONARY Investigators. Five-year outcomes after off-pump or on-pump coronary-artery bypass grafting. New Engl J Med 2016;375:2359–68. 27771985 10.1056/NEJMoa1601564

[6] Benedetto U , Caputo M, Patel NN, Fiorentino F, Bryan A, Angelini GD. Long-term survival after off-pump versus on-pump coronary artery bypass graft surgery. Does completeness of revascularization play a role? Int J Cardiol 2017;246:32–6. 28499666 10.1016/j.ijcard.2017.04.087

[7] Farkouh ME , Domanski M, Dangas GD, Godoy LC, Mack MJ, Siami FS et al; FREEDOM Follow-On Study Investigators. Long-term survival following multivessel revascularization in patients with diabetes: the FREEDOM follow-on study. J Am Coll Cardiol 2019;73:629–38. 30428398 10.1016/j.jacc.2018.11.001 PMC6839829

[8] Park SJ , Ahn JM, Kim YH, Park DW, Yun SC, Lee JY et al; BEST Trial Investigators. Trial of everolimus-eluting stents or bypass surgery for coronary disease. N Engl J Med 2015;372:1204–12. 25774645 10.1056/NEJMoa1415447

[9] Thuijs DJFM , Kappetein AP, Serruys PW, Mohr FW, Morice MC, Mack MJ et al; SYNTAX Extended Survival Investigators. Percutaneous coronary intervention versus coronary artery bypass grafting in patients with three-vessel or left main coronary artery disease: 10-year follow-up of the multicentre randomised controlled SYNTAX trial. Lancet 2019;394:1325–34. 31488373 10.1016/S0140-6736(19)31997-X

[10] Serruys PW , Morice MC, Kappetein AP, Colombo A, Holmes DR, Mack MJ et al; SYNTAX Investigators. Percutaneous coronary intervention versus coronary-artery bypass grafting for severe coronary artery disease. New Engl J Med 2009;360:961–72. 19228612 10.1056/NEJMoa0804626

[11] Nakamura M , Yaku H, Ako J, Arai H, Asai T, Chikamori T et al; Japanese Circulation Society Joint Working Group. JCS/JSCVS 2018 guideline on revascularization of stable coronary artery disease. Circ J 2022;86:477–588. 35095031 10.1253/circj.CJ-20-1282

[12] Takahashi K , Serruys PW, Fuster V, Farkouh ME, Spertus JA, Cohen DJ et al; SYNTAXES, FREEDOM, BEST, and PRECOMBAT trial investigators. Redevelopment and validation of the SYNTAX score II to individualise decision-making between percutaneous and surgical revascularisation in patients with complex coronary artery disease: secondary analysis of the multicentre randomised controlled SYNTAXES trial with external cohort validation. Lancet 2020;396:1399–412. 33038944 10.1016/S0140-6736(20)32114-0

[13] Hara H , Shiomi H, van Klaveren D, Kent DM, Steyerberg EW, Garg S et al External validation of the SYNTAX score II 2020. J Am Coll Cardiol 2021;78:1227–38. 34531023 10.1016/j.jacc.2021.07.027

[14] Kageyama S , Serruys PW, Garg S, Ninomiya K, Masuda S, Kotoku N et al; SYNTAX Extended Survival Investigators. Geographic disparity in 10-year mortality after coronary artery revascularization in the SYNTAXES trial. Int J Cardiol 2022;368:28–38. 35944766 10.1016/j.ijcard.2022.08.013

[15] Ono M , Serruys PW, Garg S, Kawashima H, Gao C, Hara H et al; for the SYNTAX Extended Survival Investigators. Effect of patient-reported preprocedural physical and mental health on 10-year mortality after percutaneous or surgical coronary revascularization. Circulation 2022;146:1268–80. 35862109 10.1161/CIRCULATIONAHA.121.057021

[16] Taggart DP , Benedetto U, Gerry S, Altman DG, Gray AM, Lees B et al; Arterial Revascularization Trial Investigators. Bilateral versus single internal-thoracic-artery grafts at 10 years. N Engl J Med 2019;380:437–46. 30699314 10.1056/NEJMoa1808783

[17] Benedetto U , Puskas J, Kappetein AP, Brown WM 3rd, Horkay F, Boonstra PW et al Off-pump versus on-pump bypass surgery for left main coronary artery disease. J Am Coll Cardiol 2019;74:729–40. 31395122 10.1016/j.jacc.2019.05.063

[18] Quin JA , Wagner TH, Hattler B, Carr BM, Collins J, Almassi GH et al Ten-year outcomes of off-pump vs on-pump coronary artery bypass grafting in the department of veterans affairs: a randomized clinical trial. JAMA Surg 2022;157:303–10. 35171210 10.1001/jamasurg.2021.7578 PMC8851363

[19] Stone GW , Kappetein AP, Sabik JF, Pocock SJ, Morice MC, Puskas J et al; EXCEL Trial Investigators. Five-year outcomes after PCI or CABG for left main coronary disease. N Engl J Med 2019;381:1820–30. 31562798 10.1056/NEJMoa1909406

[20] Park DW , Ahn JM, Park H, Yun SC, Kang DY, Lee PH et al; PRECOMBAT Investigators. Ten-year outcomes after drug-eluting stents versus coronary artery bypass grafting for left main coronary disease: extended follow-up of the PRECOMBAT trial. Circulation 2020;141:1437–46. 32223567 10.1161/CIRCULATIONAHA.120.046039

