# Supplementary material for: Impact of on-pump and off-pump coronary artery bypass grafting on 10-year mortality versus percutaneous coronary intervention
Source: Eur J Cardiothorac Surg. 2023 Jun 22;64(2):ezad240. doi: 10.1093/ejcts/ezad240 (PMC10693439; doi:10.1093/ejcts/ezad240)
Supplement: ezad240_Supplementary_Data [file ezad240_supplementary_data.docx]

**Supplemental material**

**1. Supplement for Method**

**Statistical analysis**

The primary estimand was the difference in hazard of death over 10 years in those treated with on-pump CABG compared to those treated with PCI in patients with de novo 3VD and/or LMCAD and who would be treated with on-pump CABG given a choice between on-pump and off-pump CABG. This is the so-called Average Treatment Effect on Treated (ATT) (1). The analytical approach was to initially fit a model for the probability of receiving on-pump CABG (as opposed to off-pump CABG), within the CABG-randomized arm, based on a set of baseline variables. This model would then be used to predict the probability of patients in the PCI-randomized arm receiving on-pump CABG had they been randomized to CABG. The inverse of these probabilities was the Inverse Probability of Treatment Weight (IPTW). The probability of treatment model fitted was a mixed-effects logistic regression model with the following fixed effects: age, creatinine clearance, medically treated diabetes (with or without insulin use), current smokers, peripheral vascular disease (PVD), chronic obstructive pulmonary disease (COPD), left ventricular ejection fraction (LVEF), disease type (LMCAD or 3VD), and anatomical SYNTAX score. A weighted Cox proportional hazard model was then fitted using all patients in the PCI arm and all patients in the CABG arm were confirmed as receiving On-Pump CABG. These factors are all integrated into the SYNTAX score II 2020, a comprehensive risk score predicting all-cause mortality at 10 years (2-9).

Sensitivity analyses were also performed, considering additional potential confounding factors; site distribution of the chosen CABG procedure (on-pump or on-pump), sex, and hypertension**.** Instead, creatinine clearance which has the largest missing value in the initial model was removed (Table S1). To avoid large IPTW values, only data from the sites which treated at least one patient with off-pump and on-pump CABG within the CABG-randomized arm were used. A clustering effect for the site was included along with the same set of baseline covariates used in IPTW model.

**Table S1. Confounding factors used for adjustment and the number of patients with missing value**

| Factors for adjustment | Missing number of patients (%) |
| --- | --- |
| Creatinine clearance | 132 (7.5) |
| Ever smoker | 5 (0.3) |
| Ejection fraction (categorical) | 28 (1.6) |
| SYNTAX score | 11 (0.6) |

**2. Supplement for Results**

**Post-procedural medication after revascularization**

At discharge, antiplatelet therapy was lowest in the on-pump CABG group (90.6%), while 5 years after revascularization, it was lowest in the off-pump CABG group (84.5%). During the first 5-years, statin prescriptions were highest in the PCI group (86% at discharge, 83.4% at 5-year follow-up), and whilst in the on-pump CABG group, they were lowest at discharge (74.2%) they had caught up with the PCI group at 5 years (86%). The same trend was seen in the prescription rate in the angiotensin-converting enzyme inhibitor and/or angiotensin II receptor blocker in on-pump CABG (41% at discharge and 71.9% at 5 years), causing achievement of optimal medical therapy (29.8% at discharge and 45.8% at 5 years). In the off-pump group, the statin prescription rate stayed around 75% over the first 5 years.

**Table S2. Medications at discharge and 5 years after index procedures.**

| Factor | on-pump | off-pump | PCI |
| --- | --- | --- | --- |
| OMT discharge (%) | 216 (29.8) | 58 (45.3) | 453 (50.4) |
| OMT 5y (%) | 274 (45.3) | 40 (41.2) | 358 (47.3) |
| APT discharge (%) | 657 (90.6) | 124 (96.9) | 885 (98.8) |
| APT 5y (%) | 529 (87.4) | 82 (84.5) | 692 (91.4) |
| ACEI/ARB discharge (%) | 355 (49.0) | 74 (57.8) | 600 (67.0) |
| ACEI/ARB 5y (%) | 435 (71.9) | 61 (62.9) | 539 (71.2) |
| Beta-blocker discharge (%) | 560 (77.2) | 111 (86.7) | 728 (80.6) |
| Beta-blocker 5y (%) | 444 (73.4) | 71 (73.2) | 565 (74.6) |
| Statin discharge (%) | 538 (74.2) | 97 (75.8) | 777 (86.0) |
| Statin 5y (%) | 520 (86.0) | 72 (74.2) | 631 (83.4) |

Missing patient information: 4 (0.2%)

Abbreviations: OMT, optimal medical therapy; APT, antiplatelet therapy; ACE, angiotensin-converting enzyme inhibitor; ARB, angiotensin II receptor blocker.

**Cumulative 10-year mortality stratified by SYNTAX score**

The cumulative survival curve presented in Figure 3A was stratified by 3 groups according to the anatomical SYNTAX score (SS) at baseline; Low SS (SS≤22), intermediate SS (22<SS<33), and high SS (SS≥33).

**Figure S1. Cumulative 10-year mortality stratified by SYNTAX score**

**
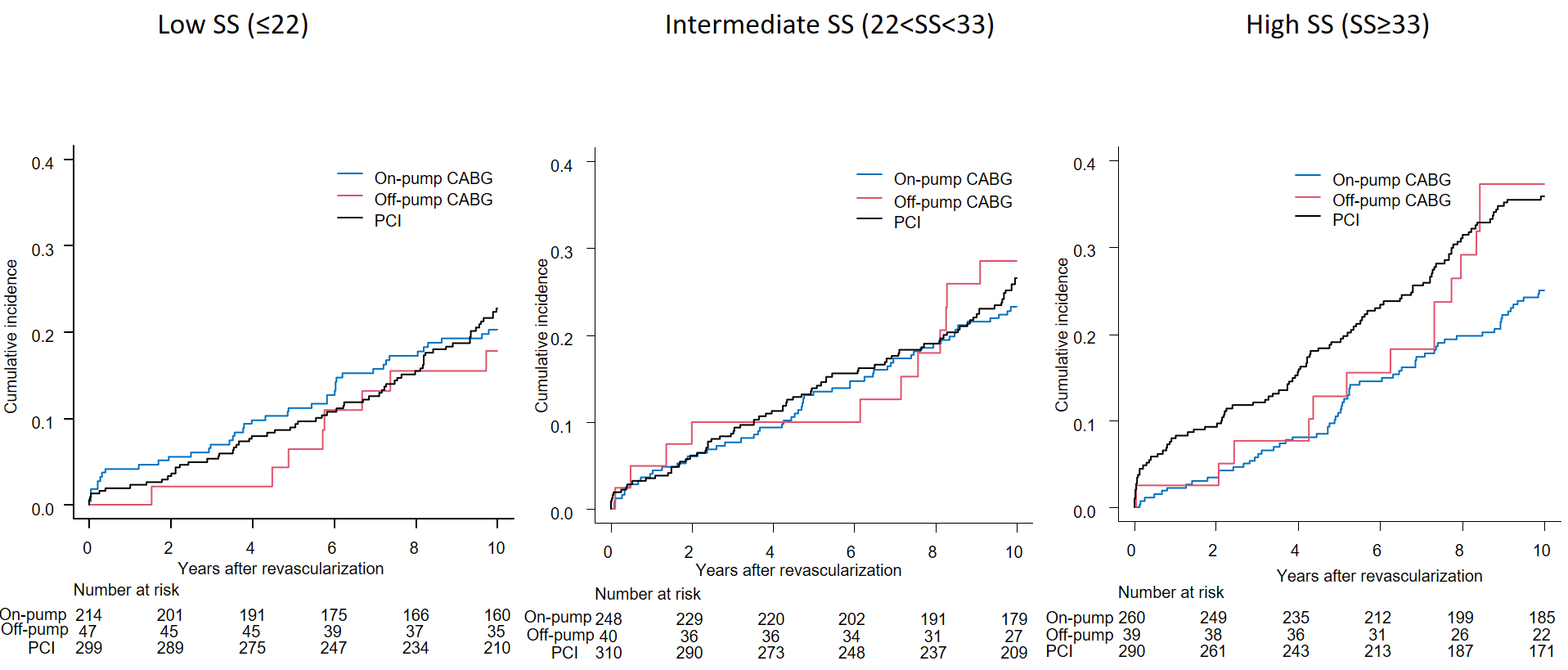
**

Abbreviations: CABG, coronary artery bypass grafts; PCI, percutaneous coronary intervention.

**Cumulative 5-year MACCE rate after revascularization**

Major adverse cardiac and cerebrovascular events (MACCE) were defined as the combination of clinical events as follows and reported as a secondary endpoint at 5 years; all-cause death, myocardial infarction, stroke, and all-cause revascularization and its components, stent thrombosis, and graft occlusion.

The cumulative incidence of MACCE up to 5 years was highest in the PCI group (37.3%), followed by off-pump CABG (28.9%) and then on-pump CABG (26.0%), as presented in Figure. Cox regression analysis showed a significantly lower event rate in on-pump CABG compared to PCI (HR 0.79, 95%CI 0.57-0.82, p<0.001). The difference in MACCE between off-pump CABG and PCI was not statistically significant (HR 0.83, 95%CI 0.59-1.19, p=0.319) but numerically favored off-pump CABG.

**Figure S2. Cumulative incidence of major adverse cardiac and cerebrovascular events in 5 years.**


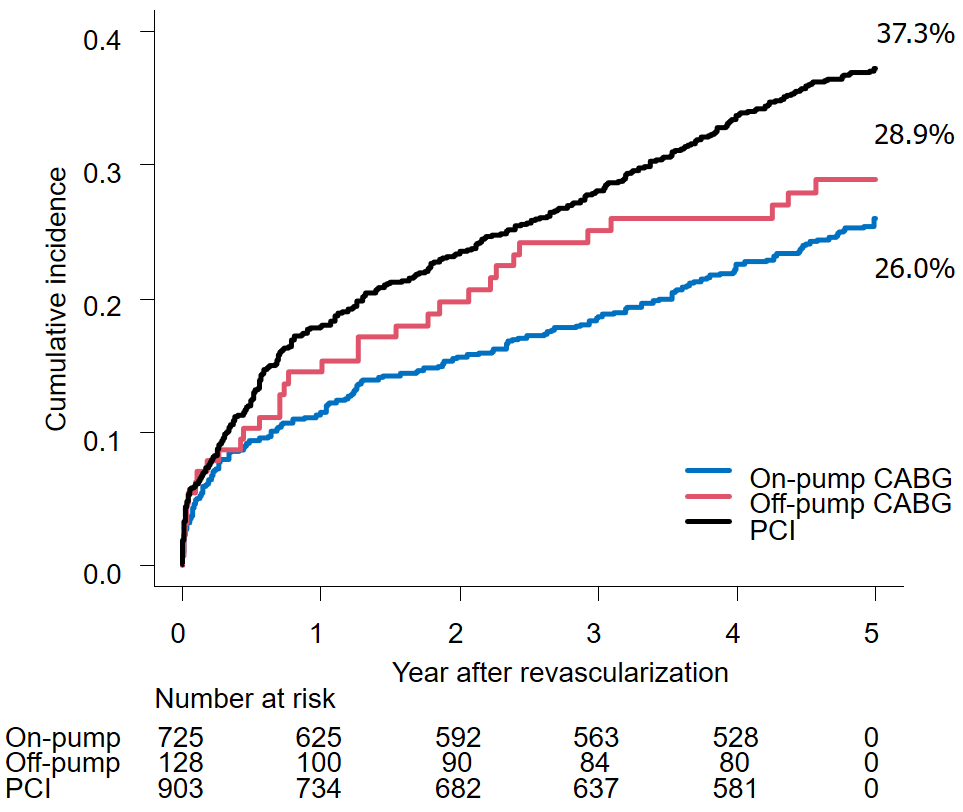


The figure showed the cumulative incidence of major adverse cardiac events for up to 5 years among the on-pump CABG, off-pump CABG, and PCI groups.

Abbreviations: CABG, coronary artery bypass grafts; PCI, percutaneous coronary intervention.

**Sensitivity analysis for the adjusted 10-year mortality**

In the sensitivity analysis of inverse probability weights, we excluded data from sites which randomly allocated patient(s) to only PCI or CABG. Considering statistical power and the missing value of the factors included in SYNTAX score II 2020, we eliminated creatinine clearance. Instead, we added sex and hypertension as potential confounding factors. Anemia can also be a factor, but we did not include it due to the number of missing values (13.9%).

Patient population after adjustment (n=1596, number of events=404) and baseline characteristics are presented in table S3.

The obtained hazard ratio of on-pump CABG against PCI is 0.66, 95% confidence interval 0.52-0.83, p=0.0003.

**Table S3. Patient population and baseline characteristics after adjustment considering the disparity of on-pump and off-pump CABG procedure and inverse propensity weighing**


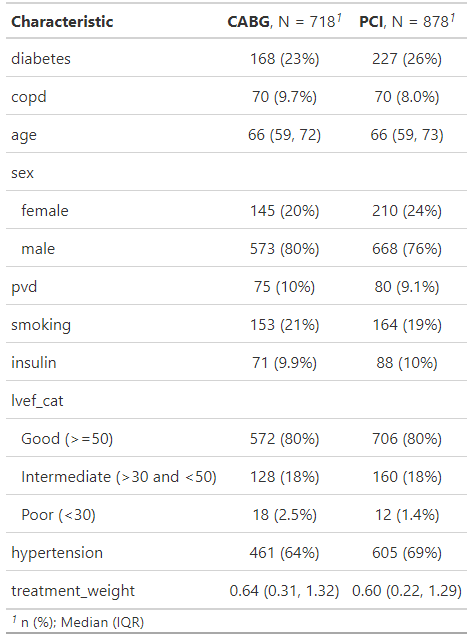


Abbreviations: CABG, coronary artery bypass graft; PCI, percutaneous coronary intervention; copd, chronic obstructive pulmonary disease; pvd, peripheral vascular disease, lvef_cat, categorical left ventricular ejection fraction.

**Individual prediction of 10-year mortality as per revascularization strategies and predicted absolute risk difference**

The predicted individual absolute risk differences (ARD) between surgical or PCI in all-cause mortality following each patient were ranked in descending order according to the predicted PCI minus predicted CABG mortality and shown by scatter plots. (10,11). The dots in the scatter plots were connected using locally estimated scatterplot smoothing (LOESS) curves. The observed mortality of treatments in individuals was calculated by the mortality of the nearest 10% population after ranking and then fitted by the LOESS curve (10-12). The external validation of the SYNTAX score II 2020 in the CREDO-Kyoto registry has shown that an individual predicted ARD in all-cause death at 5 years of <4.5% and ≥4.5% offers a sensible cut-off for “equipoise of PCI and CABG” or “CABG better,” respectively (11)..

Figure S2 shows scatter plots of individual predicted mortalities after CABG or PCI according to the SYNTAX score II 2020 and the individual observed 10-year mortality of on-pump CABG and PCI. The individual scatter plots of predicted and observed mortality are interconnected by LOWESS curves. Basically, predicted and observed mortality was superimposed in both revascularization strategies. The predicted and observed mortalities curves indicate that more than two-thirds of the randomized population would have been appropriately treated with CABG. Notably, the on-pump CABG prediction overestimated the observed mortality, especially in the low ARD population.

**Figure S3. Individual predicted and observed mortality with LOWESS curves**


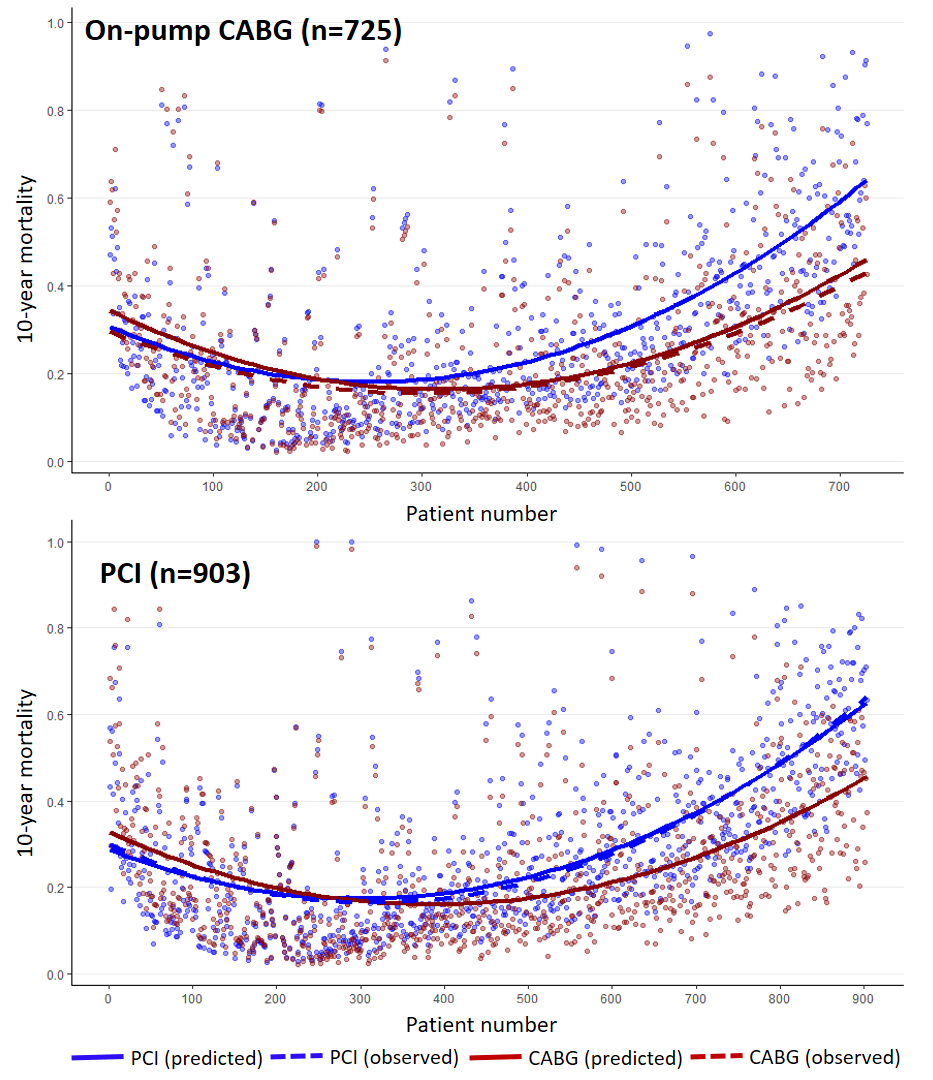


The figures present individual differences between the predicted mortality (individual scatterplots and solid smoothing curves) and the observed mortality (dashed smoothing curves) both in CABG (red) and PCI (blue). The upper panel is for the group allocated to on-pump-CABG and the lower panel is for the group allocated to PCI. The patients in the group were sorted by ARD (predicted mortality of PCI - predicted mortality of CABG). The larger ARD, which means the more to the right the patient comes, the more suitable the patient is for surgery.

Abbreviations: CABG, coronary artery bypass grafts; PCI, percutaneous coronary intervention.

[reference]

1. Imbens GW. Nonparametric estimation of average treatment effects under exogeneity: A review. Rev Econ Stat 2004;86:4-29.
2. Wang RT, Tomaniak M, Takahashi K et al. Impact of chronic obstructive pulmonary disease on 10-year mortality after percutaneous coronary intervention and bypass surgery for complex coronary artery disease: insights from the SYNTAX Extended Survival study. Clin Res Cardiol 2021;110:1083-1095.
3. Hara H, Kawashima H, Ono M et al. Impact of preprocedural biological markers on 10-year mortality in the SYNTAXES trial. Eurointervention 2022;17:1477-+.
4. Ono M, Serruys PW, Hara H et al. 10-Year Follow-Up After Revascularization in Elderly Patients With Complex Coronary Artery Disease. Journal of the American College of Cardiology 2021;77:2761-2773.
5. Ono M, Kawashima H, Hara H et al. Impact of Body Composition Indices on Ten-year Mortality After Revascularization of Complex Coronary Artery Disease (From the Syntax Extended Survival Trial). Am J Cardiol 2021;151:30-38.
6. Takahashi K, Serruys PW, Gao C et al. Ten-Year All-Cause Death According to Completeness of Revascularization in Patients With Three-Vessel Disease or Left Main Coronary Artery Disease Insights From the SYNTAX Extended Survival Study. Circulation 2021;144:96-109.
7. Kawashima H, Takahashi K, Ono M et al. Mortality 10 Years After Percutaneous or Surgical Revascularization in Patients With Total Coronary Artery Occlusions. Journal of the American College of Cardiology 2021;77:529-540.
8. Wang RT, Takahashi K, Garg S et al. Ten-year all-cause death following percutaneous or surgical revascularization in patients with prior cerebrovascular disease: insights from the SYNTAX Extended Survival study. Clin Res Cardiol 2021;110:1543-1553.
9. Kawashima H, Serruys P, Ono M, McEvoy J, Onuma Y. Impact of Optimal Medical Therapy on 10-Year Mortality After Coronary Revascularization. Journal of the American College of Cardiology 2021;78:B49-B49.
10. Takahashi K, Serruys PW, Fuster V et al. Redevelopment and validation of the SYNTAX score II to individualise decision making between percutaneous and surgical revascularisation in patients with complex coronary artery disease: secondary analysis of the multicentre randomised controlled SYNTAXES trial with external cohort validation. Lancet 2020;396:1399-1412.
11. Hara H, Shiomi H, van Klaveren D et al. External Validation of the SYNTAX Score II 2020. Journal of the American College of Cardiology 2021;78:1227-1238.
12. Ninomiya K, Serruys PW, Garg S et al. Predicted and Observed Mortality at 10 Years in Patients With Bifurcation Lesions in the SYNTAX Trial. JACC Cardiovasc Interv 2022;15:1231-1242.
